# Myosin IIB Activity and Phosphorylation Status Determines Dendritic Spine and Post-Synaptic Density Morphology

**DOI:** 10.1371/journal.pone.0024149

**Published:** 2011-08-26

**Authors:** Jennifer L. Hodges, Karen Newell-Litwa, Hannelore Asmussen, Miguel Vicente-Manzanares, Alan Rick Horwitz

**Affiliations:** 1 Department of Cell Biology, University of Virginia School of Medicine, Charlottesville, Virginia, United States of America; 2 Ramon y Cajal Program, Universidad Autonoma de Madrid School of Medicine, Hospital de la Princesa, Madrid, Spain; Northwestern University Feinberg School of Medicine, United States of America

## Abstract

Dendritic spines in hippocampal neurons mature from a filopodia-like precursor into a mushroom-shape with an enlarged post-synaptic density (PSD) and serve as the primary post-synaptic location of the excitatory neurotransmission that underlies learning and memory. Using myosin II regulatory mutants, inhibitors, and knockdowns, we show that non-muscle myosin IIB (MIIB) activity determines where spines form and whether they persist as filopodia-like spine precursors or mature into a mushroom-shape. MIIB also determines PSD size, morphology, and placement in the spine. Local inactivation of MIIB leads to the formation of filopodia-like spine protrusions from the dendritic shaft. However, di-phosphorylation of the regulatory light chain on residues Thr18 and Ser19 by Rho kinase is required for spine maturation. Inhibition of MIIB activity or a mono-phosphomimetic mutant of RLC similarly prevented maturation even in the presence of NMDA receptor activation. Expression of an actin cross-linking, non-contractile mutant, MIIB R709C, showed that maturation into a mushroom-shape requires contractile activity. Loss of MIIB also leads to an elongated PSD morphology that is no longer restricted to the spine tip; whereas increased MIIB activity, specifically through RLC-T18, S19 di-phosphorylation, increases PSD area. These observations support a model whereby myosin II inactivation forms filopodia-like protrusions that only mature once NMDA receptor activation increases RLC di-phosphorylation to stimulate MIIB contractility, resulting in mushroom-shaped spines with an enlarged PSD.

## Introduction

Dendritic spines are the primary post-synaptic sites of excitatory neurotransmission in the brain [1]. They are highly dynamic structures that develop from exploratory, filopodia-like processes into a compact, mushroom-shaped structure with a highly organized post-synaptic density (PSD) located at the tip [2], [3]. The PSD contains cell adhesion proteins, glutamate receptors, cytoskeletal molecules, and a complex membrane-associated, cytoplasmic signaling network [4], [5], [6]. Appropriate spine density, morphology, and PSD organization are critical for the neuronal function that underlies learning and memory [7], [8]. As such, a diverse spectrum of learning and memory disorders exhibit dendritic spine abnormalities, including neurodevelopmental disorders, such as autism, Down's syndrome, non-syndromic mental retardation, neurodegenerative diseases, like Alzheimer's, and psychoses, such as schizophrenia [9], [10].

Despite the importance of proper spine morphology and PSD organization, the structural and regulatory mechanisms that organize them are not understood. Recent evidence implicates the polymerization and organization of actin in spine organization, although how it does this is unclear [11], [12]. Myosin IIB (MIIB), the predominant non-muscle myosin II isoform found in brain, contributes to actin organization in most cell types through its cross-linking and contractile properties and is implicated in spine morphology [13], [14], [15]. MIIB activity is regulated by phosphorylation on residues Thr18 and/or Ser19 in its regulatory light chain (RLC); simultaneous phosphorylation on both residues promotes maximal myosin ATPase activity and formation of large actin bundles [14], [16], [17]. We have previously identified a signaling cascade that functions through RLC phosphorylation to regulate spine density [18]. More recent evidence points to MIIB as a potentially important regulator of the spine dynamics underlying learning and memory [15], [18], [19]. In particular, short-term inhibition of MIIB activity induces immature filopodia-like spines and results in a corresponding disruption of long-term potentiation (LTP) and memory acquisition [15], [19]. While the importance of MIIB seems clear, the mechanism by which it shapes spine morphology is unknown.

In addition to spine morphology, proper organization of the PSD is also important for synaptic signaling, as PSD size is related to spine head area and directly correlated with synaptic strength [20], [21]. While many molecules that reside in the PSD have been identified, much less is known about the mechanisms that determine its morphology and organization [4], [6]. The PSD is now thought to be dynamic and undergo rapid fluctuations in morphology [22], [23]. Several proteins within the PSD scaffold reportedly interact with the actin cytoskeleton [5], [24], raising the possibility that actin organization may underlie PSD morphology. The dramatic effect of MIIB on actin organization points to a likely role for it in the organization of the PSD and regulation of synaptic plasticity.

In this study, we dissect the contributions of MIIB activity to spine morphology and PSD organization during maturation and in response to stimuli. We find that MIIB activity restricts the formation of nascent protrusions on dendrites. However, MIIB activity subsequently mediates spine maturation, with RLC T18, S19 di-phosphorylation required for mature, compact spines. This maturation is mediated by the contractile activity of MIIB since an actin-cross linking, contractile-deficient mutant of MIIB, MIIB-R709C, does not promote maturation. Stimulation induced maturation of spines also requires di-phosphorylated RLC. MIIB also plays a central role in PSD organization. When inhibited, it creates elongated PSDs localized away from the spine tip; however, when fully active, it drives PSD compaction and localization to the spine tip. Thus, MIIB activity determines spine formation and orchestrates the spine and PSD morphologies that underlie post-synaptic plasticity.

## Results

### Myosin IIB Regulates Spine Morphology and Dynamics

**Figure 1:**
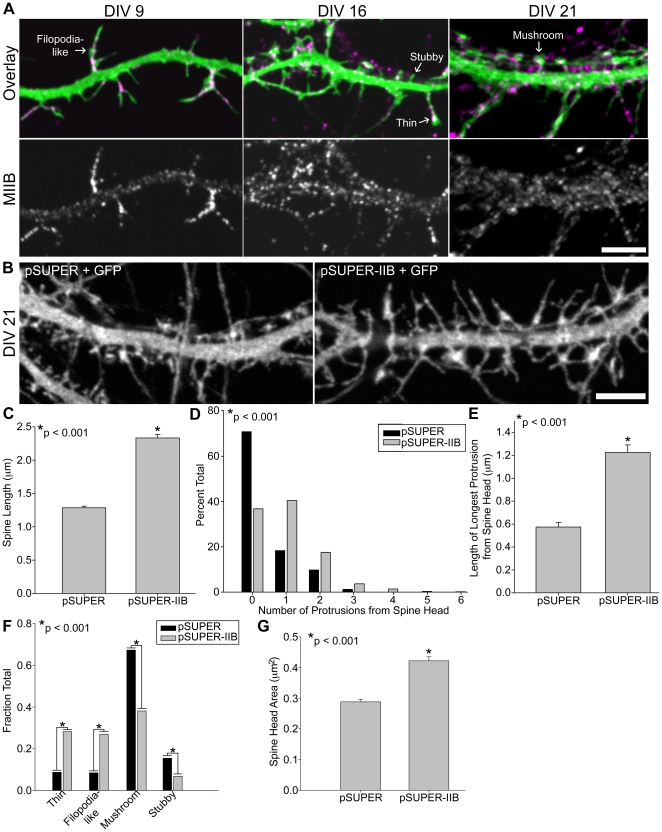
Inhibition of myosin IIB activity increases the number and length of filopodia-like protrusions. **A**) Hippocampal neurons transfected with GFP at DIV 6 were fixed and immunostained for endogenous MIIB at DIV 9, 16, and 21. Arrows point to different spine morphology types. **B**) Hippocampal neurons were co-transfected at DIV 6 with GFP and either an shRNA vector against MIIB (pSUPER-IIB) or a control empty vector (pSUPER). Neurons were fixed at DIV 21 and scored for (**C–G**) changes in spine length, branching number and length, morphology and head area. Knockdown of MIIB in hippocampal neurons causes a ∼2-fold increase in spine length, C. Knockdown of MIIB causes a large increase in the number of protrusions branching from the spine head. Spine heads were identified by morphology and localization of PSD-95. Note the small fraction of spines that contain protrusions branching from the spine head in the controls, D. MIIB knockdown produces many long protrusions branching from the spine head, which results in spine head positioning away from the spine tip, E. MIIB knockdown creates an increase in the fraction of thin (long protrusions with small head at tip) and filopodia-like spines (long protrusions without a spine head) with a concomitant decrease in the fraction of mushroom and stubby spines, F. Spine heads present in MIIB knockdown neurons are larger in area, G. For each quantification, 512 spines from 23 control neurons and 619 spines from 36 MIIB knockdown neurons were analyzed. Error bars represent SEM. p-values were derived using the Mann-Whitney test (C, D, E, G) and Chi-square test (F). Scale bar = 5 µm for all panels.

MIIB localizes to dendritic protrusions of various morphologies, including filopodia-like protrusions, as well as thin, stubby and mushroom-shaped spines (Fig. 1A). Chronic inhibition of MIIB by shRNA knockdown does not change spine density detectably (∼1.2 spines/µm dendrite for both day *in-vitro* (DIV) 21 control and MIIB-deficient neurons) [15]. Instead, it produces longer spines as measured from base to tip (including protrusions emanating from the spine head) (Fig. 1 B–C) [15], [25]. Spine heads were identified as focal expansions, which contain a PSD (PSD-95 immunostaining not shown). Noticeably, there is an increase in the number of long protrusions branching from MIIB-deficient spine heads, resulting in the spine head positioned away from the spine tip (Fig. 1 B, D, E). At DIV 21, control neurons predominantly display mushroom-shaped spines, consisting of a large bulbous spine head on top of a short spine neck. However, MIIB knockdown neurons display significantly less mushroom-shaped spines and more filopodia-like protrusions than controls (note: mushroom-shaped spines with emanating protrusions were classified as “mushroom”) (Fig. 1B, F). While these MIIB-deficient spine heads exhibit a significantly larger area than controls, they are often more elongated in shape (Fig. 1B, G). Thus, MIIB is required for spines to develop and maintain a mushroom-shape.

**Figure 2:**
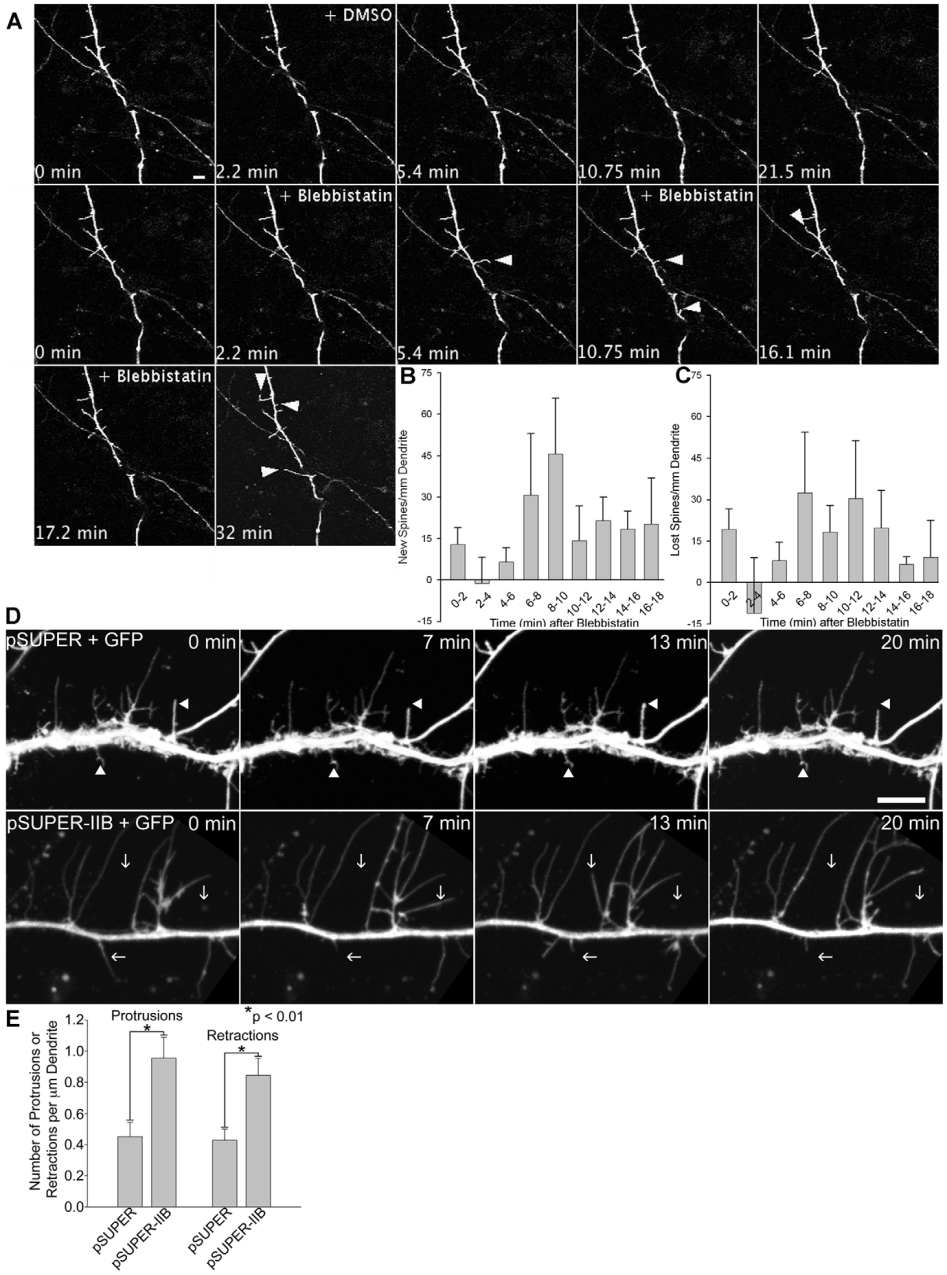
Inhibition of myosin IIB activity affects spine dynamics. **A**) A DIV 7 cortical neuron expressing DsRed2 was locally micropipetted with either DMSO or 100 µM blebbistatin at the indicated times. Note the increase in the fraction of spines that appear and extend in response to blebbistatin. Arrowheads indicate either nascent or elongating spines as shown in Video S1. Scale bar = 5 µm. **B–C**) Quantification of new spine formation (**B**) or loss of spines (**C**) following blebbistatin micropipetting (micropipetting of 5 different cortical neurons). The number of new or lost spines is corrected for the number of new or lost spines observed prior to micropipetting, i.e. the control period. **D–E**) Time-lapse confocal imaging was performed on DIV 13–14 hippocampal neurons co-expressing GFP and either an shRNA vector against MIIB or a control empty vector. Scale bar = 5 µm. Spines from MIIB knockdown neurons extend and retract more frequently (arrows) than spines in control neurons (arrowheads), D. MIIB knockdown increases the frequency of spine protrusion and retraction, E. Note the unusual length of the protrusions in the MIIB knockdown neurons. Quantification in (E) is based on 3 MIIB knockdown neurons and 5 control neurons each acquired for 15 minutes. Error bars represent SEM. *p<0.01, Mann-Whitney test.

To monitor the acute effects of MIIB inhibition on spine dynamics, we used time-lapse confocal imaging of local application of blebbistatin using a micropipette. Nascent spines emerge and protrude in response to the local application of blebbistatin (Fig. 2A and Video S1), showing that local MII inhibition leads to formation of new protrusions (Fig. 2B). However, blebbistatin micropipetting also increased spine retraction (Fig. 2C and Video S1), demonstrating that inhibition of MIIB activity does not disrupt spine pruning, but promotes the dynamic assembly and disassembly of spines. Similarly, in MIIB knockdown neurons, we observed that protrusions extend and retract more frequently and were substantially longer than those in the corresponding controls (Fig. 2D, E and Videos S2, S3). Despite their length, these protrusions are not *de novo* dendrites, as post-imaging fixation and immunostaining reveal actin-rich structures that do not contain the dendrite marker, MAP2 (data not shown).

### Myosin IIB is required for Spine Maturation in Response to NMDA Receptor Stimulation

**Figure 3:**
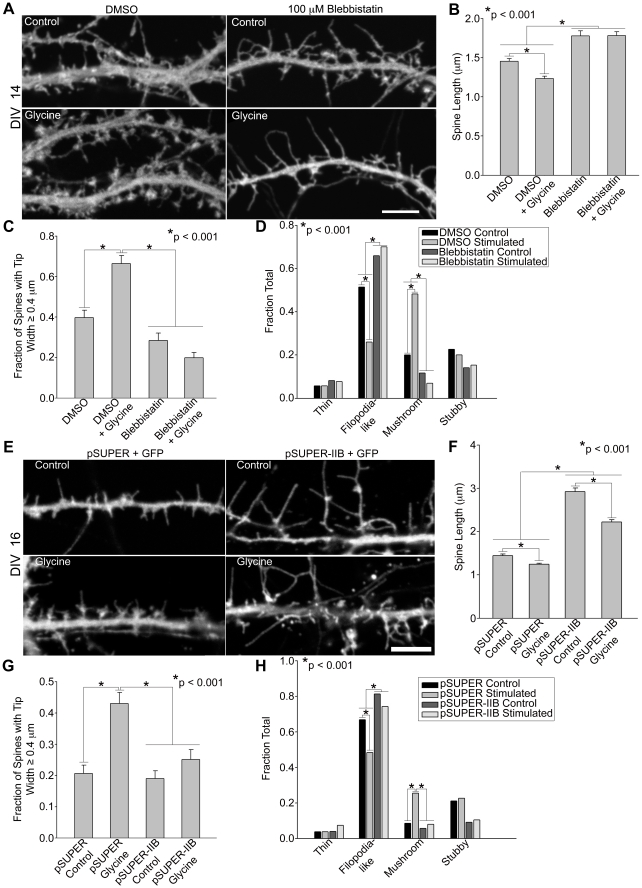
Inhibition of myosin IIB activity prevents spine morphological changes in response to NMDA receptor activation. **A, E**) When MIIB is inhibited using blebbistatin (A) or MIIB knockdown (E), spines do not shorten or assume a “mushroom” morphology in response to glycine. Hippocampal neurons were transfected on DIV 6 with GFP or co-transfected with GFP and either an shRNA vector against MIIB or a control empty vector. Neurons were treated with glycine on DIV14 (in the presence of DMSO or blebbistatin, A) or DIV16 (MIIB knockdown or empty vector control, E) to activate NMDA receptors. **B**–**D, F–H**) Quantification of spine morphology in response to MIIB inhibition and glycine stimulation. Blebbistatin (B) or MIIB knockdown (F) prevents spine shortening in response to glycine stimulation and increases spine length compared to controls; note some decrease in spine length in the knockdown in response to glycine. Fraction of spines with a large head, spine tip width ≥ 0.4 µm, increases in response to glycine stimulation but is prevented by blebbistatin (C) or MIIB knockdown (G). In the presence of blebbistatin (D) or MIIB knockdown (H), glycine does not increase the fraction of mushroom-shaped spines in contrast to stimulated controls. For each condition, 530–895 spines from 15–21 neurons were analyzed. Error bars represent SEM. *p<0.001, Mann-Whitney test (B, F), t-test (C, G), Chi-square test (D, H). Scale bar = 5 µm for all panels.

Since MIIB inhibition creates filopodia-like protrusions and inhibits spine development into compact, mushroom-shaped structures, we hypothesized that MIIB also mediates the acute, activity-induced morphology changes that underlie spine maturation. To test this, we selectively activated synaptic NMDA receptors with the co-agonist glycine and assayed for morphological changes indicative of spine maturation, including decreased spine length and increased spine tip width (i.e., mushroom-shaped) [26]. At DIV 14–17, neurons display many immature filopodia-like spines, allowing us to observe an accelerated, acute maturation response to stimulation. Glycine stimulation of control neurons promotes extensive maturation, including spine shortening and spine tip enlargement, resulting in the appearance of numerous mushroom-shaped spines (Fig. 3). In contrast, acute inhibition of MIIB with blebbistatin prevented both spine shortening and increased spine tip width; instead, spines persisted as filopodia-like projections even when stimulated with glycine (Fig. 3A–D). However, shRNA knockdown of MIIB did not prevent spine shortening in response to glycine, but did prevent an increase in spine tip width (Fig. 3E–G). Thus, shRNA knockdown of MIIB also leads to the persistence of filopodia-like protrusions (Fig. 3H). Together these results demonstrate that MIIB mediates the morphological transition from immature filopodia-like protrusions into mature mushroom-shaped spines.

### Myosin IIB-mediated Contractility Underlies Spine Maturation

**Figure 4:**
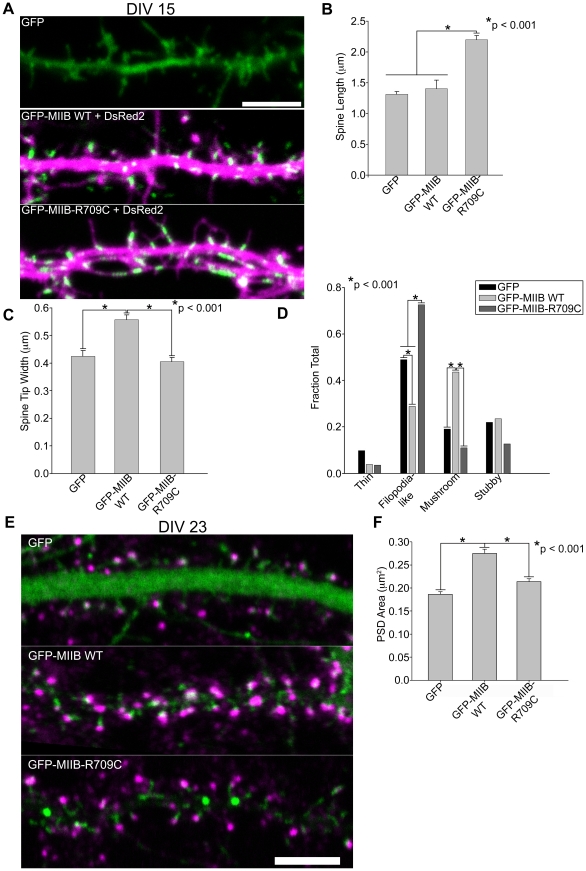
Myosin contractility promotes spine maturation. **A**) Hippocampal neurons were co-transfected at DIV 6 with DsRed2 and either GFP, GFP-MIIB WT (*wild type*), or GFP-MIIB-R709C (an actin-binding but contractile-deficient mutant) and fixed at DIV 14 or 15. Note the increased length of the non-contractile mutant and increase in mushroom-shaped spines in the cells expressing ectopic MIIB. **B–D**) Spine length, measured via DsRed2, is significantly longer in neurons expressing GFP-MIIB-R709C but is not different between GFP control and neurons expressing GFP-MIIB WT, B. Spine head width, visualized using cytoplasmic DsRed2, is greater in neurons expressing GFP-MIIB WT; but there is no difference in the spine head width of neurons expressing GFP-IIB-R709C and GFP control neurons, C. The fraction of mushroom shaped spines is greater in neurons expressing GFP-MIIB WT; whereas the fraction of filopodia-like spines is greater in neurons expressing GFP-MIIB-R709C, D. **E–F**) The PSD area increases in DIV 21–23 neurons expressing WT-MIIB, but not in the controls or neurons expressing R709C. For each condition, 424–582 spines from 6–15 neurons were analyzed. Error bars represent SEM. *p<0.001, Mann-Whitney test (B, C, F), Chi-square test (D). Scale bar = 5 µm for all panels.

MIIB organizes actin filaments by two mechanisms: it cross-links to form actomyosin bundles, and it also moves antiparallel filaments in an ATPase-dependent manner, thereby contracting them [14]. Overexpression of *wild type* (WT) MIIB accelerates spine maturation into a mushroom-shape, suggesting that MIIB-mediated contractility enhances spine maturation (Fig. 4A–D). To determine whether contractility, per se, is sufficient to create mushroom-shaped spines, we expressed a mutant, MIIB R709C, which has inhibited ATPase activity but is locked in an actin-bound state. This mutant incorporates into actomyosin bundles with high effective affinity and promotes actomyosin bundling, but not contraction [27], [28]. When MIIB-R709C is expressed in hippocampal neurons, it leads to the persistence of filopodia-like spines (Fig. 4A, D), even into later stages of neuronal development (data not shown). It also induces a two-fold longer spine length when compared to WT MIIB- or GFP-expressing controls (Fig. 4 A, B). Furthermore, WT-MIIB, but not MIIB-R709C, increased PSD size (Fig. 4E, F), which correlates with spine head volume and LTP [29]. Finally, WT-MIIB, but not MIIB-R709C, induces a significant (p<0.001) increase in the number of post-synaptic sites per µm dendrite (1.29 PSDs/µm dendrite for WT-MIIB, 0.81 PSDs/µm dendrite for GFP, and 0.75 PSDs/µm dendrite for MIIB-R709C). These results suggest that MIIB contractility mediates spine and PSD maturation.

### Differential Myosin Regulatory Light Chain (RLC) Phosphorylation Dictates Distinct Spine Morphologies

**Figure 5:**
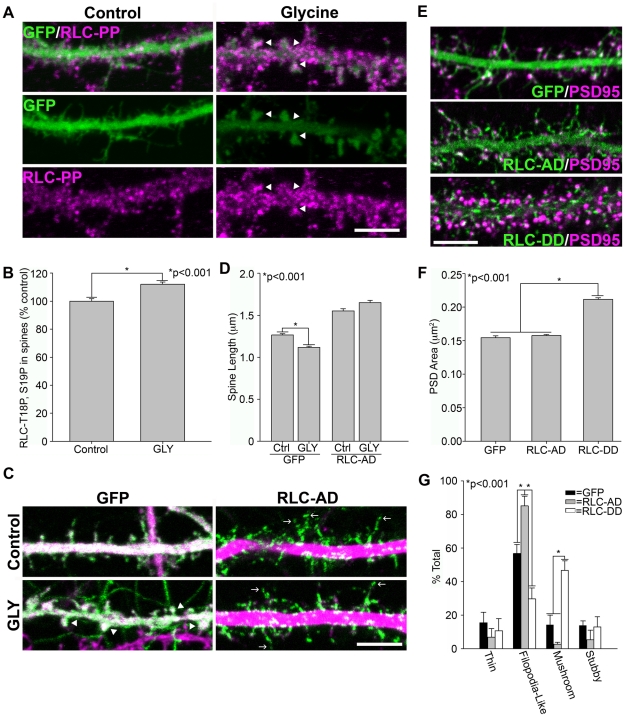
RLC T18, S19 di-phosphorylation mediates spine maturation. **A**) Glycine-activation of NMDA receptors stimulates spine maturation and increases RLC-T18, S19 di-phosphorylation in spines (arrowheads indicate increased RLC-T18, S19 ∼P in glycine-stimulated spines). DIV 21 neurons expressing GFP were chronically treated with the NMDA receptor antagonist AP-5 to inhibit spine maturation. Neurons were acutely stimulated by AP-5 withdrawal and the addition of 200 µM glycine, while control neurons were continuously treated with AP-5. **B**) Quantification of spine-associated RLC-T18, S19 di-phosphorylation by staining reveals a significant increase following NMDA receptor activation. 706 spines from 7 neurons were analyzed for AP5 controls and 843 spines from 8 glycine stimulated neurons. **C**) RLC-AD inhibits spine maturation in response to glycine activation of NMDA receptors. DIV 21 neurons were treated as described in (A) and immunostained for the dendrite marker, MAP-2 (magenta). **D**) RLC-AD prevents spine shortening in response to glycine (4C, arrows). We analyzed 2032 spines from 12 AP-5-treated GFP neurons, 1698 spines from 15 glycine stimulated GFP neurons, 1017 spines from 7 AP-5-treated RLC-AD neurons, 1116 spines from 8 glycine-stimulated RLC-AD neurons. **E**) RLC-AD expression creates filopodia-like spine precursors, while RLC-DD contracts spines into a mushroom-shaped morphology with increased PSD area. Neurons between DIV 21–33 expressing either GFP, RLC-AD GFP or RLC-DD GFP were fixed and immunostained for the PSD marker, PSD-95. **F**) RLC-DD significantly increases PSD area in comparison to GFP or RLC-AD. PSD measurements are from neurons between DIV 21–33. We analyzed 442 PSDs from 4 GFP neurons, 2204 PSDs from 16 RLC-AD neurons, and 2167 PSDs from 15 RLC-DD neurons. **G**) RLC-DD expression increases the percentage of mushroom-shape spines, while RLC-AD increases the percentage of filopodia-like spines. Spine morphology distribution of a representative culture is shown. Error bars represent SEM. *p<0.001, Mann Whitney test (B,D,F), t-test (G). Scale bar = 5 µm for all panels.

MIIB localizes to both immature filopodia-like protrusions as well as mature mushroom-shape spines (Fig. 1A). How MIIB activity is regulated to determine spine morphology is unclear. In fibroblasts, simultaneous RLC phosphorylation on residues T18 and S19 increases MIIB activity and creates front-back polarity [16], [30]. We therefore asked whether RLC phosphorylation regulates post-synaptic MIIB activity to create mature mushroom-shaped spines. In response to NMDA receptor activation by glycine, we stained for di-phosphorylated RLC (T18, S19), and observed a significant increase (Fig. 5 A-B). To determine whether RLC-T18∼P, S19∼P di-phosphorylation is necessary for spine maturation, we activated NMDA receptors with glycine in neurons expressing RLC-T18A, S19D (RLC-A, D), which mimics mono- but prevents di-phosphorylation [16]. While control neurons matured into a mushroom-shaped spine, RLC-A,D prevented spine maturation; instead they persisted as filopodia-like protrusions (Fig. 5 C–D). In contrast, expression of a di-phosphomimetic mutant, RLC-T18D,S19D (RLC–D,D) increased spine maturation and PSD area when compared to GFP or RLC-AD expressing neurons (Fig. 5 E–G). Therefore, while mono-phosphorylation inhibits spine maturation and PSD enlargement, RLC di-phosphorylation is necessary for and promotes it.

### Rho Kinase (ROCK) regulates RLC T18, S19 di-phosphorylation and spine maturation

**Figure 6:**
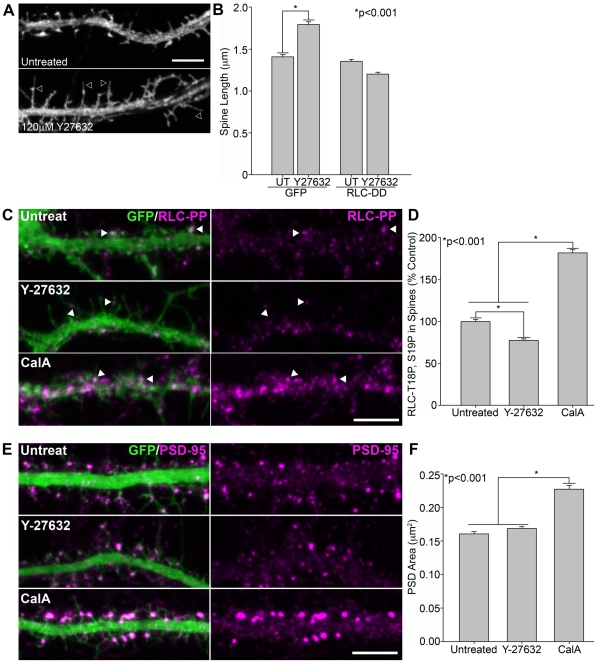
ROCK regulates spine morphology through RLC-T18, S19 di-phosphorylation. **A**) ROCK inhibition (Y-27632) produces filopodia-like spines (arrowheads). DIV14 neurons expressing GFP were treated with 120 µM Y-27632 for 2 hours or left untreated as a control. **B**) RLC-DD prevents the increase in spine length with Y-27632 We analyzed 1199 spines from 13 GFP untreated neurons, 1056 spines from 9 GFP neurons treated with Y-27632, 1142 spines from 6 RLC-DD untreated neurons, and 809 spines from 8 RLC-DD neurons treated with Y-27632. **C**) Y-27632 decreases endogenous RLC-T18, S19 di-phosphorylation concomitant with the formation of filopodia-like spines. In contrast, inhibition of myosin light chain phosphatase with calyculin A (CalA), increases RLC-T18, S19 di-phosphorylation. Arrowheads indicate spine-associated RLC-PP. Neurons were treated with 100 µM Y-27632 for 2 hours or 20nM calyculin A for 20min or left untreated. **D**) Y-27632 decreases the levels of spine-associated RLC-PP staining; whereas calyculin A increases it. We analyzed 855 spines from 10 untreated neurons, 901 spines from 9 Y-27632-treated neurons, and 989 spines from 9 calyculin A-treated neurons. **E–F**) Calyculin A increases PSD area in comparison with untreated or Y-27632-treated neurons. Neurons were treated as in C. We analyzed 499 PSDs from 10 untreated neurons, 519 PSDs from 9 Y-27632-treated neurons, and 452 PSDs from calyculin A-treated neurons. Error bars represent SEM. *p<0.001, Mann-Whitney test. Scale bar = 5 µm.

ROCK is a kinase that increases RLC phosphorylation on T18 and S19 both directly and indirectly through inhibition of myosin light chain phosphatase [31], [32]. We therefore determined whether ROCK regulates post-synaptic RLC di-phosphorylation and spine morphology. Neurons treated with the ROCK inhibitor, Y-27632, showed long filopodia-like spines with an increased length (Fig. 6 A–B) and similar to those observed when RLC di-phosphorylation is inhibited by expression of RLC-AD (Fig. 5) [33]. In contrast, inhibition of myosin light chain kinase, another RLC kinase, did not increase spine length (data not shown) [34]. Furthermore, expression of the di-phosphomimetic RLC-D,D mutant superseded the effects of Y-27632 on spine length, suggesting that RLC is a major post-synaptic target of ROCK activity (Fig. 6B). Using an antibody specific for di-phosphorylated RLC T18P, S19P, we observed an ∼20% decrease in the post-synaptic levels of di-phosphorylated RLC with Y-27632, coincident with an increase in spine length (Fig. 6 C–D). Calyculin A, which inhibits myosin light chain phosphatase [35] increased RLC-P,P and induced the formation of mushroom-shaped spines with enlarged PSDs (Fig. 6 C–F). Thus, post-synaptic regulation of RLC di-phosphorylation underlies spine maturation.

### Myosin IIB Regulates Post-Synaptic Density Organization

**Figure 7:**
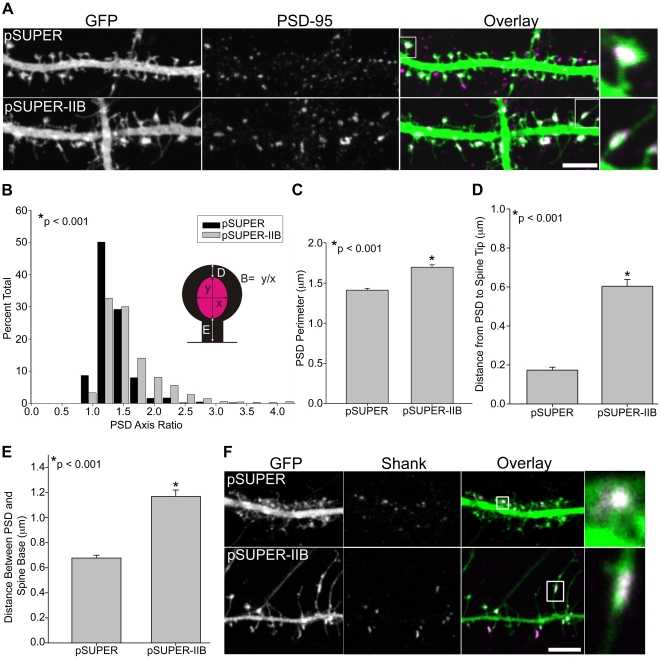
Myosin IIB regulates post-synaptic density morphology. **A**) Myosin IIB knockdown alters PSD morphology and positioning. Hippocampal neurons were co-transfected on DIV 6 with GFP and either an shRNA vector against MIIB or a control empty vector and fixed and immunostained for endogenous PSD-95 at DIV 21. **B**) The PSD axis ratio (B) is expressed as the long axis (y) of each PSD divided by the short axis (x). The PSD axis ratio is significantly greater in neurons with MIIB knocked down. **C**) shRNA knockdown of MIIB increases the PSD perimeter. **D**) Distance from PSD-95 to the spine tip (D in diagram) is significantly greater in neurons with MIIB knocked down. **E**) Distance from PSD-95 to the spine base (E in diagram) is significantly greater in neurons with MIIB knocked down. For each condition, 524–738 spines of 10–14 neurons were analyzed. **F**) Immunostaining for Shank confirms the elongated PSD morphology in response to MIIB knockdown. Error bars represent SEM. *p<0.001, Mann-Whitney test. Scale bar = 5 µm.

The PSD is a highly ordered, yet dynamic structure, undergoing continual variations in morphology [22]. We therefore asked whether actomyosin activity regulated the size, shape, or location of the PSD in the spine. To study PSD morphology, we stained for the PDZ-containing synaptic scaffold protein PSD-95, which is a canonical PSD marker that appears early during PSD formation [36]. Whereas control spines exhibit a compact, round, or slightly elliptical PSD, MIIB knockdown spines displayed an elongated PSD with larger perimeters (Fig. 7 A–C). Furthermore, in control cells, PSD-95 localizes mainly to the spine tip; however, in MIIB-deficient neurons, the elongated PSD localizes away from the spine tip and base, toward the center of the filopodia-like spine (Fig. 7 D, E). Similar results were observed using another PSD marker, shank (Fig. 7F) [24], suggesting that MIIB controls the morphology of the PSD globally, rather than through specific effects on some of its constituents.

## Discussion

Non-muscle myosin II plays a major role in the organization of actin filaments and dictates the diverse morphologies and directional movement of various cell types. These include the apical constriction of epithelial cells, nuclear positioning, orientation of the microtubule-organizing center, Golgi and the contractile ring of dividing cells, and polarization of migrating fibroblasts [14]. Of the MII isoforms, MIIB is the predominant one found in hippocampal neurons, and its activity and effective affinity for actomyosin filaments is regulated by RLC [13], [14]. Previous studies have implicated MIIB as a target of a signaling pathway that is mutated in non-syndromic mental retardation and in spine development and memory formation [15], [18], [19], [37]. We now address the mechanisms by which MIIB acts on spines and show that differential MIIB activity determines where spines form, creates diverse post-synaptic spine morphologies, and mediates the morphology, size, and positioning of the PSD. It also mediates the changes in spine morphology in response to stimuli. Thus, MIIB emerges as a major downstream regulator of the component processes underlying post-synaptic plasticity, and implicitly, learning and memory.

Spine maturation consists of three stages: emergence of protrusions along the dendritic shaft, spine elongation, and maturation into a mushroom-shape [3]. Our results demonstrate that differential MIIB activity mediates and coordinates these diverse stages of spine development. Highly branched and dynamic spines emerge along the dendritic shaft and proceed to develop into the long dendritic protrusions that characterize immature spines, which persist in the absence of full, i.e., di-phosphorylated RLC, MIIB activation. This suggests that MIIB normally functions to restrict membrane protrusion and branching [38], [39]. It also suggests that the elongation of filopodia-like protrusions occurs in the absence of strong MIIB contractile activity. Several observations support this hypothesis. Myosin IIB inhibition or knockdown produces numerous long filopodia that do not mature [15]. In addition, the contractile-deficient myosin IIB mutant, R709C, cross-links but does not contract actin and results in persistently long spines. Similarly, inhibition of RLC T18, S19 di-phosphorylation by expressing RLC T18A, S19D or inhibiting ROCK activity using Y-27632 similarly produces filopodia-like spine precursors; however we cannot exclude contributions from other ROCK targets, like LIMK1 [33], [40], [41].

Excitatory stimulation increases PSD size, which directly correlates with synaptic strength and leads to long-term potentiation [7], [29], [42]. MIIB determines PSD positioning as well as its morphology. When MIIB is inhibited, the PSD becomes elongated and is no longer at the spine tip. An analogous change is seen in migrating fibroblasts, where large central adhesions tend to disperse when MII activity is inhibited [43], [44], [45]. In addition, increased myosin IIB activity via RLC T18, S19 di-phosphorylation, enlarges both the PSD and fibroblast adhesions [16]. In this context, the combination of crosslinking and contraction induced by MII activity, likely serves to cluster the numerous PDZ- and SH3-domain containing actin binding proteins found within the PSD [46], [47], [48]. MIIB-generated forces could also increase PSD size by inducing conformational changes in PSD components that present new binding sites for the recruitment of additional molecules, as also reported in fibroblasts [49], [50].

During post-synaptic development, changes in spine morphology correlate with changes in PSD organization and synaptic signaling. Specifically, maturation of spines into a mushroom-shape and PSD enlargement at the spine tip enhances the synaptic signaling that underlies learning and memory formation [7]. Our findings show that myosin IIB coordinates the spine and PSD morphological changes that occur in response to excitatory stimulation. Furthermore, differential regulation of MIIB activity through RLC phosphorylation states switches spine and PSD shape from filopodia-like spine precursors with smaller PSDs to mature mushroom-shape spines with larger PSDs. Thus, myosin IIB serves as a critical regulator of post-synaptic plasticity, consistent with the observation that myosin IIB is necessary for memory formation [19].

Our observations and previous literature lead to a model for the role of MIIB in spine formation and maturation. Spines form in regions of inactive MIIB and can extend into long filopodia-like structures in the absence of high MIIB activity. The most likely mechanism for this formation and extension is due to localized activation of Rac. The GIT1/PIX/PAK complex, which contains the Rac-activator PIX and Rac-effector PAK, is one mechanism by which Rac activation is localized to generate spines [18], [51]. These filopodia-like spines are highly dynamic and protrude and retract frequently; since MIIB is not required for this activity, it is likely that this arises largely from actin polymerization and depolymerization. In contrast, the maturation into a compact, mushroom-shaped structure requires MIIB contractile activity; however, Arp2/3-driven actin polymerization may contribute as well to drive spine head expansion, in analogy with the broad protrusions it mediates in migrating fibroblasts [52], [53], [54]. Finally, MIIB may also serve to localize signals that affect spine morphology and function, such as GEFs that mediate Rac activity, e.g., ß-PIX and Kalirin-7, or other mechanoresponsive molecules that regulate signaling in other cell types [18], [55], [56]. Our holistic view of the effect of myosin II on the component processes of post-synaptic development provides the framework for the identification of critical therapeutic targets, such as ROCK, for the treatment of learning and memory disorders.

## Materials and Methods

### Antibodies and reagents

Postsynaptic density-95 (PSD-95) monoclonal antibody was purchased from Santa Cruz Biotechnology (Santa Cruz, CA) and used at ratio of 1∶100 for immunostaining. Non-muscle myosin heavy chain II-B polyclonal antibody was obtained from Covance (Emeryville, CA) and used at a ratio of 1∶1000. A polyclonal antibody against phosphorylated RLC-T18, S19 was purchased from Cell Signaling Technologies and used at a ratio of 1∶100-1∶200 (Danvers, MA). Secondary anti-mouse and anti-rabbit antibodies conjugated to Alexa488, 568 and 647 were from Invitrogen. Blebbistatin, Calyculin A, and Y-27632 were purchased from Calbiochem (La Jolla, CA) and used at the concentrations indicated in the figures. Tetrodotoxin and strychnine were purchased from Sigma and reconstituted in dH_2_O.

### Plasmids

The shRNA knockdown vector for MIIB has been described elsewhere [27]. GFP-MIIB was a gift from Robert S. Adelstein [57]. RNAi-insensitive GFP-MIIB and GFP-MIIB-R709C mutants have been described previously [27]. The 3′-UTR encompassing 1500nt's was cut out of both GFP-MIIB and GFP-MIIB-R709C vectors using XmaI restriction enzyme. The 1.5 kb DNA piece was ligated into the 9 kb vector backbone and sequenced to verify correct orientation of the insert. PSD-95-GFP was a gift from David Bredt [58]. RLC-GFP constructs (WT, DD) were kindly provided by Kathleen Kelly (National Cancer Institute, Bethesda, MD), and RLC-AD-GFP was generated as previously described [16].

### Neuronal culture and transfection

Low-density hippocampal cultures were prepared from E19 rat embryos as described previously [59]. All experiments were carried out in compliance with the Guide for the Care and Use of Laboratory Animals of the National Institutes of Health and approved by the University of Virginia Animal Care and Use Committee (Protocol Number: 2884). Neurons were plated on glass coverslips coated with 1 mg/ml poly-L-lysine at an approximate density of 70 cells/mm^2^ and were transfected using a modified calcium phosphate precipitation method as described previously [51]. Cortical neurons were nucleofected with DsRed2 as described by [60], and plated on poly-L-lysine coated imaging dishes. DIV 5–12 cortical neurons were micropipetted with 100 µM-1 mM blebbistatin for 10 msec-1 sec with 5psi pressure using an IM 300 Microinjector from Narishige International USA, Inc. (East Meadow, NY). For the chemical stimulation experiments involving knockdown or inhibition of MIIB (Fig. 3), DIV14–17 neurons were removed from the glia-feeder layer and placed in 1X Mg^2+^-free extracellular solution containing 15 mM NaCl, 0.5 mM KCl, 0.2 mM CaCl_2_, 3 mM glucose, 1 mM Hepes, 0.5 µM tetrodotoxin, and 1 µM strychnine, pH7.4 [26]. Stimulated neurons are treated with 200 µM glycine and incubated at 35°C, 5% CO_2_ for 3 min. The solution is removed and replaced with 1X Mg^2+^-free extracellular solution with tetrodotoxin and strychnine and incubated at 35°C, 5% CO_2_ for 20 minutes before fixation. For inhibition of MIIB activity with blebbistatin, neurons were pre-treated for 30 minutes and throughout the protocol with either 100 µM blebbistatin or a corresponding volume of DMSO as a control. Alternatively (Fig. 5), neurons were chronically treated with 100 µM of the NMDA receptor antagonist, AP-5, from DIV 6-21 to inhibit NMDA receptor activation and spine maturation. Neurons were then stimulated by AP-5 withdrawal and addition of 200 µM glycine, while control neurons continued in the presence of AP-5 (200 µM), as described by others [61], [62].

### Immunocytochemistry

Neurons were fixed in PBS containing 4% formaldehyde, methanol-free, ultra-pure EM grade (Polysciences, Inc., Warrington, PA) with 4% sucrose for 20 min at room temperature and permeabilized with 0.2% Triton X-100 for 10 min. Alternatively, for PSD-95 and RLC-T18P, S19P staining, neurons were simultaneously fixed and permeabilized in 2% formaldehyde with 4% sucrose for 10 min at room temperature and then with cold methanol for 10 min at −20°C. After blocking with 20% goat serum/PBS for one hour at room temperature, the neurons were incubated with the appropriate antibodies in 5% goat serum/PBS for one hour at 37°C. RLC-PP staining was performed in PBS only. Coverslips were mounted with Vectashield mounting media (Vector Laboratories, Burlingame, CA).

### Imaging and analysis

Confocal images were collected on an Olympus Fluoview 1000 microscope (IX81 base) equipped with a 60X/1.35 NA (oil) UPLSAPO 60X objective (Olympus). Green probes (GFP and Alexa488) were excited using the 488 nm laser line of a multi Ar laser; red probes (DsRed2 and Alexa568) were excited with the 543 nm laser line of a He-Ne laser; the far-red probe Alexa647 was excited with the 635 nm line of an LD laser. Fluorescence emission was collected using the following dichroic mirror/filter combinations: SDM560/BA505–525 (GFP), SDM640/BA560–620 (DsRed2, Alexa568 and RhodamineX) and BA655–755 (Alexa647). Two-color fluorescence images of Alexa488 (GFP)/Alexa568 (RhodamineX/DsRed2) were collected in a Z-stack and in sequential mode. Images were acquired using Fluoview software (Olympus). Spine length, width, PSD-95 long and short axis, area, and perimeter were quantified using Image J software. Statistical analysis was performed using Sigma Plot 11. Spine morphologies were defined as either filopodia-like, thin, mushroom, or stubby [3]. Filopodia-like spines are long and thin without a spine head, whereas thin spines contain a small head at the spine tip. Mushroom-shaped spines are shorter with a large spine head atop a neck. Stubby spines are short protrusions, either thin or wide, with no discernable neck. Statistical analysis of spine morphology in Figures 1, 3 and 4 were performed with SAS 9.2.

## Supporting Information

Video S1 **Local blebbistatin micropipetting increases spine formation.** Blebbistatin, but not DMSO, increases spine formation and extension (arrowheads). Equal volumes of blebbistatin and DMSO were used. Video S1 corresponds to still images in Fig. 2A. Images were acquired every ∼6 seconds using confocal microscopy. 20 frames/sec shown.
(AVI)

Video S2 **Spine dynamics of control neurons.** Time-lapse confocal imaging was performed on DIV14 hippocampal neurons co-expressing GFP and a control empty vector. Video S2 corresponds to still images in top row of Fig. 2D. Images were acquired every 1-minute using confocal microscopy. 3 frames/sec shown.
(AVI)

Video S3 **MIIB knockdown increases spine dynamics.** Spines from MIIB knockdown neurons extend and retract more frequently than spines in control neurons (S2). Note the increased length of spine extensions in MIIB knockdown neurons. Time-lapse confocal imaging was performed on DIV14 hippocampal neurons co-expressing GFP and an shRNA vector against MIIB. Video S3 corresponds to still images in bottom row of Fig. 2D. Images were acquired every 1-minute using confocal microscopy. 3 frames/sec shown.
(AVI)
